# Gratitude Heals: State Gratitude Weakens the Objectification-Social Pain Link

**DOI:** 10.3390/bs15111452

**Published:** 2025-10-25

**Authors:** Junjie Qiu, Jiaxin Shi, Zhansheng Chen

**Affiliations:** 1 Guangdong Provincial Key Laboratory of Development and Education for Special Needs Children, Lingnan Normal University, Zhanjiang 524048, China; qiujjie@lingnan.edu.cn; 2 Department of Psychology, School of Educational Sciences, Lingnan Normal University, Zhanjiang 524048, China; 3 Faculty of Psychology, South China Normal University, Guangzhou 510631, China; 4 Department of Psychology, The University of Hong Kong, Hong Kong SAR, China; chenz@hku.hk

**Keywords:** gratitude, objectification, social pain, well-being

## Abstract

From the targets’ perspective, objectification is the process of being perceived and treated as mere instruments without human qualities. We argue that objectified people would experience more social pain and that the state of gratitude could weaken the link between objectification and painful feelings. Three studies (N = 927) confirmed our hypotheses. Study 1 found that people experienced more social pain after recalling the objectification experience. In Study 2, the participants’ chronic objectification was positively linked to psychological pain. More importantly, participants with higher feelings of objectification reported lower pain in the gratitude condition than those in the non-gratitude condition. In Study 3, objectified people reported less social pain in the gratitude condition than in the non-gratitude condition. In sum, our research highlights the negative impacts of objectification and the power of gratitude as a valuable tool in buffering the adverse effects of objectification.

## 1. Introduction

No one would doubt the importance of social connections to our lives. Indeed, people inherently seek to establish and nurture social bonds with others, driven by deep-rooted psychological motivations (Baumeister & Leary, 1995; Deci & Ryan, 2000). However, when these bonds are threatened or severed, the resultant emotional distress, termed “social pain”, can be profound (Chen et al., 2008; Chen & Williams, 2012; Eisenberger et al., 2003). Social pain serves as an adaptive mechanism, signaling potential risks to our social ties (MacDonald & Leary, 2005). Given its foundational role in preserving the integrity of human relationships, social pain has profound implications. It can influence cognitive functions, alter behavioral patterns, and even manifest in physical symptoms (Chen et al., 2008; Vangelisti et al., 2005; Twenge et al., 2001). Previous studies have demonstrated that multiple contexts can induce social pain, such as ostracism (Eisenberger et al., 2003), personal adversity (Hudd & Moscovitch, 2021), and loneliness (Baumeister et al., 2002). In the current research, we aimed to identify another common but unexplored phenomenon that may induce social pain: objectification.

### 1.1. Objectification and Its Impact

Objectification, a pervasive occurrence in daily life (Holland et al., 2017), denotes the treatment or perception of individuals as mere objects (Nussbaum, 1995). While prior research has predominantly centered on objectification within sexual contexts (Fredrickson & Roberts, 1997; Morris et al., 2018; Holmes & Johnson, 2017), more recent studies have investigated objectification from non-sexual perspectives, encompassing working (Belmi & Schroeder, 2020), intergroup relations (Markowitz & Slovic, 2020), and general interpersonal interactions (Poon et al., 2020; Shi et al., 2023). When individuals fall victim to objectification, they are often stripped of their inherent humanity and relegated to the role of mere instruments. According to Nussbaum’s (1995) framework, objectification encompasses seven key aspects: instrumentality (seeing targets as tools for personal goals), agency deprivation (regarding targets as lacking independent action), experiential nullification (perceiving targets as devoid of emotional experiences), fungibility (treating targets as replaceable by others with similar traits), violability (perceiving targets as susceptible to intrusion), and ownership (attributing credibility to targets). Among these features, instrumentality is the central and defining aspect of objectification, distinguishing it from related constructs such as dehumanization (Orehek & Weaverling, 2017; Zhang et al., 2025). Dehumanization involves denying human qualities, either by likening people to machines (mechanistic) or to animals (animalistic). While both objectification and dehumanization deny agency and experience, objectification uniquely emphasizes instrumentality, viewing others as tools to achieve one’s goals.

The conceptualization implies that objectification is detrimental to targets due to their reduction to mere tools and the perception of their deficiency in essential human attributes (e.g., agency and experience; Andrighetto et al., 2017; Baldissarri et al., 2014; Loughnan et al., 2010). Indeed, many studies have indicated that objectification produces adverse consequences, heightened aggression (Poon et al., 2020; Shi & Chen, 2024), job burnout (Baldissarri et al., 2014), diminished feelings of belonging (Belmi & Schroeder, 2020), and poor well-being (Cheng et al., 2022). However, some studies suggest that objectification can also be beneficial. For instance, relationships that involve a higher degree of instrumentality are often perceived as stronger and closer than those without such elements (Fitzsimons & Fishbach, 2010). More broadly, people tend to value and appreciate others who help them achieve their personal goals (Converse & Fishbach, 2012; Fitzsimons et al., 2015; Orehek & Weaverling, 2017). These contradictory findings make it especially important to empirically examine the psychological outcomes of objectification. To address this problem, we examined whether objectification leads to social pain.

### 1.2. Objectification and Social Pain

We argue that objectification can trigger social pain through the deprivation of fundamental psychological needs. When individuals are objectified, they are treated as passive instruments rather than autonomous agents, which undermines their sense of control, agency, and belonging (Belmi & Schroeder, 2020; Cheng et al., 2022; Dai et al., 2023). According to self-determination theory, the frustration of such basic needs produces distress (Deci & Ryan, 2000). Moreover, research shows that sexual objectification can evoke ostracism-related feelings and threaten fundamental need satisfaction (Dvir et al., 2021). Ostracism is tightly associated with social pain (Eisenberger, 2012; Williams, 2009). Together, these theoretical and empirical insights support our argument that objectification will be positively associated with social pain.

Interestingly, a prevailing assumption seems to suggest that objectified individuals are less likely to experience pain. As eloquently posited by Dworkin (2000, p. 30), “When objectification occurs, the person is depersonalized”. Indeed, an object traditionally lacks the capacity for pain; similarly, objectified individuals are presumed to possess reduced pain sensitivity as their holistic human experience is negated. Loughnan et al. (2010) explored this assumption empirically, discovering that participants in their experiment assigned more quantities of pain-inducing tablets to objectified individuals. However, these discussions and findings have predominantly centered on the perspective of those enacting objectification, neglecting the viewpoint of the objectified victims. To the best of our knowledge, no study has directly examined the link between objectification and psychological pain from the vantage point of the targets themselves. Consequently, our first goal was to bridge this research gap by investigating whether objectification engenders feelings of pain among those who experience it.

### 1.3. The Buffering Effect of Gratitude

Objectification may heighten feelings of social pain, underscoring the importance of identifying potential buffers of this effect. We propose that gratitude functions as one such buffer. Gratitude is commonly defined as a positive emotion that arises when individuals recognize and appreciate receiving valuable benefits, support, or kindness from others (Emmons et al., 2003; McCullough et al., 2004). Previous studies have shown many benefits of gratitude in multiple domains such as increasing well-being (Wood et al., 2009), and prosociality (Stellar et al., 2017; Nowak & Roch, 2007) and decreasing negative experiences (e.g., anxiety and stress; Kendler et al., 2003; Wood et al., 2008), aggression (DeWall et al., 2012), and objectification (Shi et al., 2023).

Our argument that gratitude buffers the objectification-social pain link can be supported by theoretical and empirical research. First, the broaden-and-build theory (Fredrickson, 2001) proposes that positive emotions such as gratitude expand cognitive and behavioral repertoires, enabling individuals to shift away from narrowed, ruminative responses to negative experiences and instead engage in adaptive coping strategies. This broadening process fosters social and psychological resources that buffer against social pain. Indeed, previous studies have shown that due to its positive valence nature, gratitude can resist multiple undesirable feelings (Emmons & McCullough, 2003; Geraghty et al., 2010; Watkins et al., 2003).

Second, the “find-remind-and-bind” theory posits that gratitude functions as a psychological gel, strengthening one’s social relationships (Algoe, 2012). In support of this notion, cross-sectional and longitudinal studies have shown that gratitude is related to robust perceived social support (Froh et al., 2009; Kong et al., 2015; Lin & Yeh, 2014; Wood et al., 2008). Since enhanced social bonds can mitigate social pain, gratitude should be able to buffer the effect of objectification on social pain.

Drawing further evidence from direct research on gratitude, people with high gratitude reported lower physical and psychological pain (Cudney et al., 2024; Froh et al., 2009; Hill et al., 2013; Yu et al., 2017; except for Emmons & McCullough, 2003). For example, early adolescents with stronger gratitude reported fewer physical symptoms including chest and stomach pain (Hill et al., 2013). However, most studies are correlational; thus, experimental research that can determine causality is in great need (see also Alkozei et al., 2018). In response, the current work aims to address this issue by manipulating gratitude. In sum, based on the theories and findings, gratitude signals positivity and social support, which should counteract the effect of objectification on social pain.

### 1.4. Overview of Research

Across three studies, we aimed to examine the relationship between objectification and social pain as well as the potential buffering role of gratitude. We first examined whether objectification leads to social pain, which lays the foundation of the subsequent studies. Study 1 tested the basic premise that objectification elicits social pain by asking participants to recall and describe an experience of being objectified. Study 2 extended this investigation by examining whether gratitude moderates the association between cumulative objectification experiences over the past month and current social pain. Finally, Study 3 used a fully experimental design to manipulate both objectification and gratitude, allowing us to test the causal buffering effect of gratitude on the immediate experience of objectification.

Given that the study was conducted in China, all materials and measures were translated into Chinese, adhering to Brislin’s (1970) established translation method, which involved a translation and subsequent back-translation carried out by two psychology master’s students. The procedures were approved by the corresponding author’s university IRB (SCNU-PSY-2023-016). All participants provided their informed consent before participating in the study. We uploaded all data and materials online (https://osf.io/s8be5/?view_only=3775097787504220b5b2fedd99fbd1ee (accessed on 24 June 2024)). We reported the sensitive power analysis for each study to determine the effect size. The power analysis was conducted by G*power 3.1, and the statistical analysis was conducted by Jamovi 2.3.28.

## 2. Study 1

Study 1 aimed to test whether objectification leads to social pain. Prior research suggests that when individuals are objectified, they are deprived of essential human attributes and fundamental needs, which contributes to heightened psychological distress (Williams, 2009; Belmi & Schroeder, 2020; Cheng et al., 2022; Dai et al., 2023). Moreover, objectified individuals often report experiences similar to ostracism (Dvir et al., 2021), a condition closely tied to social pain (Eisenberger et al., 2003). Building on these insights, we hypothesized that participants in the objectification condition would experience stronger social pain than those in the control condition. To test this hypothesis, we employed a recall-and-writing paradigm, a method widely used in prior research (e.g., Poon et al., 2020), in which the participants were asked to recall and describe a personal experience of being objectified.

### 2.1. Materials and Methods

#### 2.1.1. Participants

Study 1 was conducted in a lab where we recruited 127 Chinese undergraduates from Lingnan Normal University (97 women). Their mean age was 19.18 years (SD = 1.07, range: 16–23). All students provided informed consent and received course credits as compensation. A sensitivity analysis on this number of participants showed that we would acquire an effect size of at least d = 0.5, with α = 0.05 and power = 0.80.

#### 2.1.2. Procedure and Measures

We randomly assigned participants to one of two conditions (objectification vs. control). In each condition, participants completed the writing task on a computer in the laboratory and were given up to 10 min to finish. The experimenter ensured that all participants spent an equivalent amount of time on the task before proceeding to the subsequent measures. In the objectification condition, they read the following instructions:

Objectification refers to being perceived or treated as a tool. When others objectify you, you are only an object in their eyes. Please recall and write down an experience of when you were objectified.

Participants were instructed to write down what they did last Wednesday in the control condition.

After completing the writing task, participants reported their pain feelings using the FPS (Bieri et al., 1990; Fillingim et al., 2016). This scale includes pictures of seven faces that each indicate different social pain levels (0 = “not painful at all” to 6 = “extremely painful”). Participants were then instructed to select one face reflecting their current pain state. After responding to a few demographic questions (i.e., those on their age and gender), the participants were thanked and debriefed.

### 2.2. Results and Discussion

An independent *t*-test on painful feelings showed a significant difference between objectification vs. control conditions (Welch’s t(111.09) = 5.19, *p* < 0.01, Cohen’s d = 0.93, 95% CI [0.54, 1.30]). Participants in the objectification condition reported a greater level of pain (N = 63; M = 2.24, SD = 1.57) than those in the control condition (N = 64; M = 0.98, SD = 1.11).

These results support our hypothesis that objectification causes the targets to experience a sense of social pain. However, a limitation should be noted that a manipulation check was not included. We addressed this issue in the subsequent studies.

## 3. Study 2

Study 1 showed that objectification increases social pain. Study 2 aimed to test whether experimentally-induced gratitude could buffer the relationship between objectification experiences and social pain. According to the broaden-and-build framework (Fredrickson, 2001) and “find-remind-and-bind” theory (Algoe, 2012), gratitude can broaden coping repertoires, promote belongingness, and provide an affective signal of social support, which directly counters the negative message of objectification. We therefore hypothesized that self-reported objectification over the past month would predict stronger social pain, but that this effect would be attenuated among participants in the gratitude induction condition compared with those in the control condition. To this end, we measured the participants’ experiences of objectification, manipulated their state of gratitude, and then measured their painful feelings.

### 3.1. Materials and Methods

#### 3.1.1. Participants

We recruited 400 Chinese participants via Credamo (Mage = 31.41, SD = 8.22; 277 women). All participants were compensated approximately USD 1 upon completion. No participants were excluded as all completed the study and passed the attention check item embedded in the scale (“Please select 2 on this item”). The sensitive power analysis with α = 0.05 and power = 0.8 indicated that the current sample size could detect the minimal effect size η^2^ = 0.02.

#### 3.1.2. Procedures and Measurements

Participants were informed that they would participate in the study, which consisted of a few unrelated parts. First, the participants reported their perceived objectification during the last month, measured by a four-item scale adapted from Gruenfeld et al. (2008). The sample item was “I feel that others treated me as a tool”. Participants responded to each item on a seven-point Likert scale (1 = not at all, 7 = very often). The participants’ responses were averaged to index their feelings of objectification (α = 0.93). Afterward, the participants were randomly assigned to one of two conditions (gratitude vs. control). In each condition, the participants were instructed to complete a writing task within 5 min. In the gratitude condition, participants were asked to write a thank-you letter to someone they felt grateful to, while in the control condition, participants were asked to write down what they did last Wednesday. This manipulation method has been used in previous studies to promote people’s state of gratitude (e.g., Deichert et al., 2021; Shi et al., 2023). After writing, the participants responded to one item regarding their gratitude (“I feel grateful now.” 1 = not at all, 7 = very much so) as a manipulation check. Finally, the participants reported their pain on the Faces Pain Scale used in Study 1. Participants were then instructed to select one face reflecting their pain state. After responding to a few demographic questions (i.e., those on their age and gender), the participants were thanked and debriefed.

### 3.2. Results

Manipulation check: An independent *t*-test on the manipulation check showed that participants in the gratitude condition felt a stronger level of grateful feeling (N = 200, M = 6.17, SD = 0.98) than those in the control condition (N = 200, M = 4.24, SD = 1.83), t(398) = 13.15, *p* < 0.001, d = 1.31, 95% CI [1.08, 1.55]. The results showed that our manipulation succeeded.

Social pain: We conducted a multiple regression analysis on psychologically painful feelings to examine our hypothesis. The experimental condition was coded (1 = gratitude, 0 = control), the objectification was centered, and their interaction was created. The results showed that objectification positively predicted pain, B = 0.38, SE = 0.05, *p* < 0.001, η^2^ = 0.14, 95% CI [0.29, 0.47], whereas the main effect of gratitude was not significant, B = −0.14, SE = 0.15, *p* = 0.344, η^2^ = 0.001, 95% CI [−0.44, 0.15].

**Figure 1:**
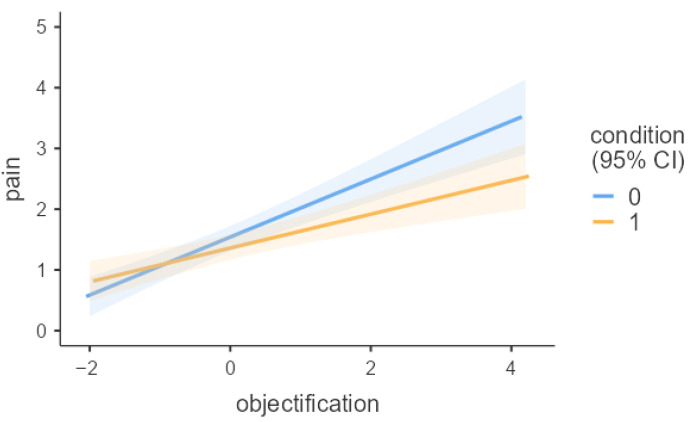
Gratitude induction moderates the relationship between objectification and social pain (Study 2). Note. Condition: 0 = control, 1 = gratitude.

More importantly, the expected interaction of objectification and gratitude emerged, B = −0.2, SE = 0.09, *p* = 0.031, η^2^ = 0.01, 95% CI [−0.38, −0.02]. The simple slope analysis indicated that participants with high levels of objectification (+1 SD) reported lower pain feelings in the gratitude condition compared with the control condition (B = −0.47, SE = 0.21, t = −2.19, *p* = 0.029, 95% CI [−0.89, −0.05]). In contrast, among participants with low (−1 SD) and medium levels of objectification, participants who felt gratitude reported pain levels similar to the control group (low: B = 0.18, SE = 0.21, t = 0.87, *p* = 0.387, 95% CI [−0.23, 0.60]; medium: B = −0.14, SE = 0.15, t = −0.95, *p* = 0.344, 95% CI [−0.44, 0.15]) (see Figure 1).

### 3.3. Discussion

The findings supported our hypothesis that the induction of gratitude could alleviate the relationship between objectification and painful feelings. Specifically, people who had more objectification experiences reported stronger painful feelings, whereas people reported lower painful feelings after writing a gratitude letter. Our findings demonstrate that cumulative objectification experiences can predict people’s momentary feelings of social pain. Importantly, gratitude induction attenuated this link. In this sense, gratitude functions as a psychological buffer, lowering the impact of past objectification on present well-being.

## 4. Study 3

Study 3 advanced the findings in two crucial ways. First, we manipulated objectification and gratitude to test whether fostering gratitude may alleviate the painful feelings stemming from objectification. Second, we used another measure to assess the participants’ pain. Using the different measures could enhance the robustness of our findings. We pre-registered Study 3 at https://aspredicted.org/LLS_G2P (accessed on 1 April 2024).

### 4.1. Materials and Method

#### 4.1.1. Participants

We recruited 400 Chinese participants (224 women; Mage = 30.59, SD = 9.45, ranging from 18–69) via Credamo to participate in this study. All participants were compensated approximately RMB 2 upon completion. As they were pre-registered, no participants were excluded as they all completed the study and passed the attention check embedded in the scale (“Please select 6 on this item”). According to a sensitive power analysis with α = 0.05, power = 0.8, this sample size could detect the minimal effect size η^2^ = 0.02.

#### 4.1.2. Procedures and Measurements

Following previous studies (e.g., Dai et al., 2023; Shi & Chen, 2024), we employed a double-randomization method to independently manipulate objectification and gratitude. In particular, we randomly assigned the participants to one of two conditions (objectification vs. control). In both conditions, they were instructed to finish an imaginary writing task within 5 min. Specifically, in the objectification condition, the participants were given the following instructions:

*Suppose that you are currently working in a business. While interacting with your colleagues, they treat you like an object. They regard you as an instrument to fulfill a particular purpose.*

*Please imagine a scenario like this and describe it in writing.*

Participants were required to imagine and write down an ordinary business day in the control condition. After that, the participants needed to complete one item (“Working here, I feel objectified”) as a manipulation check on a seven-point scale (1 = not at all, 7 = very much so). Afterward, the participants were again randomized into gratitude or non-gratitude conditions. Those in the gratitude condition were prompted to write a thank-you letter expressing their gratitude to someone, while those in the non-gratitude condition documented their activities from the previous Wednesday. Identical to Study 2, participants were instructed to complete the writing task within 5 min. Post-writing, the participants assessed their social pain using a three-item scale adapted from the Mee–Bunney Psychological Pain Assessment Scale (MBPPAS; Mee et al., 2011). The items included “Please select the number that describes your psychological pain at this moment” (1 = no pain, 5 = unbearable), “How much more psychological pain do you think you can tolerate before it becomes unbearable?” (1 = quite a bit more, 5 = already unbearable), and “Compared to the worst physical pain you can imagine, how would you rate your psychological pain at present?” (1 = no pain, 5 = much more painful). We averaged the scores from the three items to produce an index to indicate the participants’ painful feelings (α = 0.7). Finally, after the participants disclosed their gender and age, they were thanked and debriefed.

### 4.2. Results

**Figure 2:**
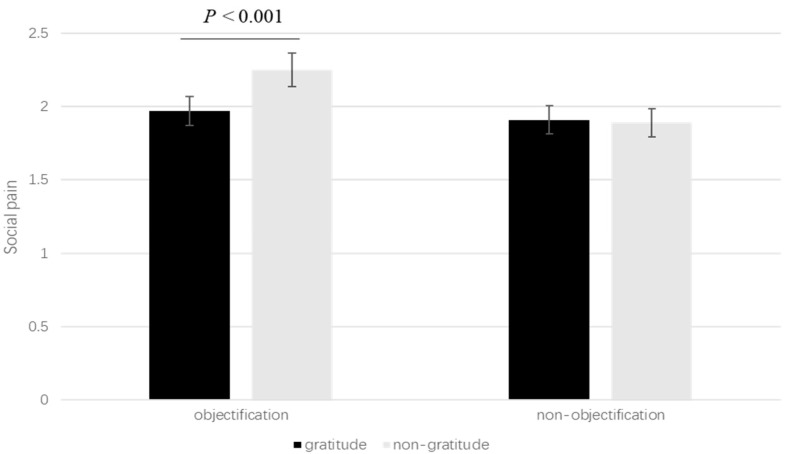
Gratitude induction buffers the effect of objectification on social pain (Study 3). Error bars indicate the 95% CI.

To explore our hypothesis that gratitude could mitigate the painful feelings after objectification, we conducted a 2 × 2 ANOVA on painful feelings, with objectification and gratitude as two factors. The results revealed a significant main effect of objectification on painful feelings, F(1, 396) = 9.46, *p* < 0.002, partial η^2^ = 0.02. Specifically, participants in the objectification condition reported a higher level of painful feelings (M = 2.11, SD = 0.76) than those in the control condition (M = 1.88, SD = 0.07). In contrast, a direct comparison between the gratitude and non-gratitude conditions did not yield a significant difference, F(1, 396) = 2.44, *p* = 0.119, partial η^2^ = 0.01. More importantly, there was a significant interaction between objectification and gratitude, F(1, 396) = 5.27, *p* = 0.022, partial η^2^ = 0.01. Specifically, the objectified people felt less pain (M = 1.97, SE = 0.07) after writing a gratitude letter than those writing a non-gratitude letter (M = 2.25, SE = 0.07), t(396) = −3.8, *p* < 0.001. No significant difference was found in the non-objectification conditions, *p* = 0.954 (see Figure 2). The results support our hypothesis that gratitude reduces painful feelings induced by objectification.

### 4.3. Discussion

The present findings provide experimental evidence supporting our hypothesis that the state of gratitude could alleviate social pain induced by objectification. Consistent with the previous findings, Study 3 showed that objectified people felt less pain after feeling gratitude. This study extended the earlier study by experimentally manipulating objectification and gratitude. Notably, we only used one item to check the effectiveness of our manipulation, which may raise concerns about the reliability. Future studies should incorporate more comprehensive manipulation checks to strengthen the validity.

## 5. General Discussion

Objectification, as perceived from the target’s vantage point, is the experience of being treated as an object rather than a human being, and it has been shown to yield detrimental consequences (Baldissarri et al., 2014; Belmi & Schroeder, 2020; Nussbaum, 1995; Obermann, 2011; Poon et al., 2020; Shi & Chen, 2024). Extending this line of work, we examined whether objectification elicits social pain—an unpleasant emotional experience that arises from actual or perceived threats to one’s sense of social connection or social value (Eisenberger, 2012)—and, if so, how this effect can be buffered.

Three studies provided convergent empirical evidence supporting our hypotheses that objectification triggers social pain and that gratitude can mitigate this relationship. Study 1 established the causal link between objectification and social pain. Study 2 showed that objectification is positively associated with social pain and that gratitude weakens this relationship. In Study 3, we manipulated the objectification conditions, demonstrating that individuals in the gratitude condition reported less social pain than those in a neutral condition when highly objectified. These consistent findings across different research designs underscore the harmful effects of objectification by revealing its association with social pain. More importantly, the results highlight the potential of gratitude to influence psychological responses following objectification.

The present findings contribute to the research on the outcomes of objectification. In line with previous studies (Cheng et al., 2022; Poon et al., 2020; Shi et al., 2023), our results consistently underscore the negative impacts of objectification within non-sexual contexts, thus advancing our understanding of the psychological repercussions of being objectified. Previous research has shown that people tend to evaluate objectified targets as less sensitive to pain because they perceive them as less than human (Loughnan et al., 2010), and even objectified individuals perceive themselves as lacking essential human characteristics including agency and experience (Loughnan et al., 2017). Our results directly affirm that objectified targets do experience psychological pain. We do not intend to suggest that our findings contradict prior research (e.g., Loughnan et al., 2010). Rather, our work adds evidence from a neglected but important perspective: that of the targets themselves. In other words, even when objectified individuals are perceived by others as lacking certain human attributes, they nonetheless feel the pain of objectification. These asymmetrical findings highlight the importance of considering both the perspectives of the objectifiers and the lived experiences of the targets in future research.

Moreover, our study accentuates the detriment of instrumental relationships. While social relationships are pivotal for psychological well-being (Baumeister & Leary, 1995), not all relationships are beneficial. Our results suggest that instrumental relationships yield adverse effects, particularly for the targets objectified as mere tools. The discourse surrounding the universal harm of instrumental relationships is contentious. Some studies suggest that objectification is harmful (Belmi & Schroeder, 2020), whereas others believe it can be beneficial (Orehek & Forest, 2016). For example, Fitzsimons and Fishbach (2010) found that people perceived instrumental others as closer. Our findings support the former view. We argue that the disparities in the results stem from two factors. First, our study concentrated on the recipients of objectification, whereas others have centered on those perpetuating objectification (Fitzsimons & Fishbach, 2010). Second, our study’s definition of objectification and that of Belmi and Schroeder (2020) encompassed a broader scope than prior studies (e.g., Orehek & Forest, 2016) that predominantly focused on instrumentality. This distinction implies that the negative consequences of objectification may not solely be attributed to instrumentalization. As noted by Nussbaum, “What is problematic is not instrumentality per se, but treating someone primarily or merely as an instrument” (Nussbaum, 1995, p. 265). Thus, the key mechanism underlying the effects of objectification may lie in whether the target is fully recognized as a person. When individuals are valued for their usefulness but still perceived as fully human, instrumental relationships may be welcomed. In contrast, when individuals are valued solely for their utility and perceived as lacking humanness, instrumental relationships become harmful. Future research should further investigate this potential moderation mechanism.

The current findings contribute to the field of gratitude by nicely dovetailing with previous studies demonstrating the benefits of gratitude. In particular, the current finding highlights the positive effect of gratitude in an intrapersonal domain. Gratitude helps foster positive states (e.g., well-being; Emmons & McCullough, 2003) and plays a pivotal role in mitigating unfavorable conditions (e.g., worry and body dissatisfaction; Geraghty et al., 2010). Building upon these insights, we extend the healing function of gratitude in attenuating psychological pain stemming from objectification. However, the mechanisms underpinning this beneficial effect remain to be elucidated. There are several proposed theories accounting for the positive effects of gratitude including the positive affect hypothesis (Watson & Naragon-Gainey, 2010), the coping hypothesis (Wood et al., 2007), and the find-remind-and-bind hypothesis (Algoe, 2012). It is conceivable that the amelioration in pain after writing a gratitude letter arises from heightened positive feelings (as per the positive affect hypothesis), a strengthened sense of social support (aligned with the find-remind-and-bind idea), or a combination of both. Future investigations are warranted to distinguish and test these hypotheses further.

Notably, while we indicate that gratitude reduces the effect of objectification on social pain, the severity of objectification in our studies was relatively mild. For example, in Study 3, the participants imagined workplace scenarios that may not reflect the extreme forms of objectification (e.g., sweatshop labor). Therefore, caution is needed when extrapolating our findings to more severe or prolonged forms of objectification. Future research should examine whether gratitude has similar protective effects in such high-intensity contexts.

Future research may continue to investigate whether gratitude can moderate other harmful outcomes of objectification. For example, a recent study suggested that objectification in the workplace leads to self-harm behavior and that the effect was lessened when the participants perceived higher alternatives in life (Dai et al., 2023). We believe that gratitude may have an identical function because gratitude provides people meaning in life that can confront self-harm (Manela, 2022; O’Dea et al., 2024).

In addition to its theoretical contributions, the present study holds practical implications. Given that perceived objectification triggers psychological distress in those being objectified, it becomes imperative for organizational managers to proactively avoid cultivating instrumental relationships with their employees, thus safeguarding their psychological well-being. Moreover, corporate culture should prioritize their employees’ individuality over their utilitarian role.

Several limitations should be acknowledged to inspire future studies. First, we only recruited Chinese participants via Credamo in the current three studies, limiting our findings’ generalization. Future studies may include more representative participants with diverse backgrounds. Second, we employed the imagination paradigm to experimentally manipulate objectification in Study 3. Although the paradigm has been successfully used to induce objectification in previous studies (e.g., Poon et al., 2020; Shi et al., 2023), it undermines the ecological validity of the findings. Future studies may replicate the current study using more natural designs such as diary studies. Third, we acknowledge that the writing task used in the control condition (i.e., describing activities from the previous Wednesday) may not fully rule out potential valence confounds. Indeed, there is debate over whether objectification can be fully explained by other social evaluations (Over, 2021; Vaes et al., 2021). Some studies suggest that objectification as well as its related concept dehumanization can be reduced to misogyny (Statman, 2024) or intergroup preference (Bunce et al., 2025), whereas other studies suggest that objectification/dehumanization constitutes a unique construct that cannot be reduced merely to negative evaluation (Bruneau et al., 2018; Shi et al., 2023) or intergroup bias (Vaes et al., 2021). Future studies could address this issue by including two types of control conditions: one involving the recall of a general negative experience, and another involving a strictly neutral task such as describing one’s route to campus or workplace. More broadly, this also raised an important issue in the field of objectification. To fully understand the unique role of objectification, researchers may investigate a more fundamental question regarding the concept of the “human” or “humanness” (see also Bunce et al., 2025). Investigating how this concept is understood is key to clearly differentiating humans from non-human species and inanimate matter. By exploring this foundational issue, researchers can more precisely identify the distinct role of dehumanization/objectification and other forms of social evaluations (e.g., negative evaluation and interpersonal harm). Fourth, we only used the self-report method to measure psychological pain. Although the self-report is frequently used, it still faces several challenges (see Hudd & Moscovitch, 2021). Thus, future researchers can test these findings by using other measures of psychological pain such as fMRI (Eisenberger et al., 2003). Finally, the present research design cannot reveal how long the effect of gratitude works on weakening the social pain triggered by objectification. It would be helpful to adopt a longitudinal design to address this limitation.

## 6. Conclusions

Our study reveals that objectification instigates feelings of psychological pain. More importantly, gratitude can reduce the impact of objectification on psychological pain. These innovative discoveries enhance comprehension of the adverse consequences of objectification, particularly from the standpoint of the recipients.

## Data Availability

The raw data supporting the conclusions of this article will be made available by the corresponding author on request.
